# Role of *yqiC* in the Pathogenicity of *Salmonella* and Innate Immune Responses of Human Intestinal Epithelium

**DOI:** 10.3389/fmicb.2016.01614

**Published:** 2016-10-10

**Authors:** Ke-Chuan Wang, Chih-Hung Huang, Shih-Min Ding, Ching-Kuo Chen, Hsu-Wei Fang, Ming-Te Huang, Shiuh-Bin Fang

**Affiliations:** ^1^Division of Pediatric Gastroenterology and Hepatology, Department of Pediatrics, Shuang Ho Hospital, Taipei Medical UniversityTaipei, Taiwan; ^2^Department of Pediatrics, School of Medicine, College of Medicine, Taipei Medical UniversityTaipei, Taiwan; ^3^Graduate Institute of Biochemical and Biomedical Engineering, National Taipei University of TechnologyTaipei, Taiwan; ^4^Institute of Biomedical Engineering and Nanomedicine – National Health Research InstitutesZhunan, Taiwan; ^5^Department of Surgery, Shuang Ho Hospital, Taipei Medical UniversityTaipei, Taiwan; ^6^Department of Surgery, School of Medicine, College of Medicine, Taipei Medical UniversityTaipei, Taiwan

**Keywords:** *yqiC*, bacterial colonization, menaquinone, *Salmonella* Typhimurium, interleukin-8, human β-defensin-3, flagella, type-1 fimbriae

## Abstract

The *yqiC* gene of *Salmonella enterica* serovar Typhimurium (*S*. Typhimurium) regulates bacterial growth at different temperatures and mice survival after infection. However, the role of *yqiC* in bacterial colonization and host immunity remains unknown. We infected human LS174T, Caco-2, HeLa, and THP-1 cells with *S*. Typhimurium wild-type SL1344, its *yqiC* mutant, and its complemented strain. Bacterial colonization and internalization in the four cell lines significantly reduced on *yqiC* depletion. Post-infection production of interleukin-8 and human β-defensin-3 in LS174T cells significantly reduced because of *yqiC* deleted in *S*. Typhimurium. The phenotype of *yqi*C mutant exhibited few and short flagella, fimbriae on the cell surface, enhanced biofilm formation, upregulated type-1 fimbriae expression, and reduced bacterial motility. Type-1 fimbriae, flagella, SPI-1, and SPI-2 gene expression was quantified using real-time PCR. The data show that deletion of *yqiC* upregulated *fimA* and *fimZ* expression and downregulated *flhD, fliZ, invA*, and *sseB* expression. Furthermore, thin-layer chromatography and high-performance liquid chromatography revealed the absence of menaquinone in the *yqiC* mutant, thus validating the importance of *yqiC* in the bacterial electron transport chain. Therefore, YqiC can negatively regulate FimZ for type-1 fimbriae expression and manipulate the functions of its downstream virulence factors including flagella, SPI-1, and SPI-2 effectors.

## Introduction

*Salmonella enterica* serovar Typhimurium (i.e., *S*. Typhimurium) is a notorious food-borne pathogen that causes gastroenteritis in animals and humans worldwide. *Salmonella* exhibit classic virulence factors, such as plasmids, toxins, fimbriae, and flagella (van Asten and van Dijk, 2005). Most virulence genes are clustered in regions distributed throughout the chromosome called SPIs. Thus far, at least 11 SPIs have been identified. SPI-1 and SPI-2, the most commonly studied SPIs, encode T3SSs that translocate the effector proteins into host cells or secrete them into the extracellular environment, thus affecting host biochemistry and cell physiology (Sabbagh et al., 2010). SPI-1 genes are involved in the key events of early disease phases, such as membrane ruﬄing through cytoskeleton manipulation (Zhou et al., 2001), virulence regulation (Raffatellu et al., 2005), and innate immunity activation, including neutrophil recruitment across the epithelium and NF-κB signaling activation (Lee et al., 2000). By contrast, SPI-2 is responsible for intracellular transport and survival, particularly in the systemic phase of a disease (Coburn et al., 2007), and for cyclooxygenase 2 induction (Uchiya and Nikai, 2004), host cytokine modulation (Uchiya and Nikai, 2005), and cell death (Knodler et al., 2005).

In *S*. Typhimurium, *yqiC* is located outside SPIs, and most of its functions are unclear, except for its contribution to bacterial survival at various temperatures and to host survival in mice (Carrica et al., 2011). In our preliminary study, a *yqiC* transposon mutant of *S.* Typhimurium, created using transposon-directed insertion-site sequencing, could not colonize HEp-2 cells (Fang, 2011). Thus, *yqiC*, a non-SPI gene, is responsible for bacterial colonization and subsequent invasion during early bacteria–host interactions. However, this finding requires affirmation. In the process of bacterial colonization, fimbrial (Baumler et al., 1996) or adhesin-based (Lambert and Smith, 2008) adherence and subsequent bacterial invasion contribute to *Salmonella* virulence, accompanied by host neutrophil recruitment and transcellular signaling that orchestrate the pathogenicity (McCormick et al., 1993; Pace et al., 1993). In *Salmonella*, fimbrial adhesins include Fim, Pef, Bcf, Sti, Stf, Saf, Stb, Csg, Stc, Std, Lpf, Stj, and Sth (McClelland et al., 2001), whereas non-fimbrial adhesins include ShdA, MisL, SadA, SiiE, and BapA (Wagner and Hensel, 2011). However, YqiC is not classified in either of these categories. The role of *yqiC* in the early pathogenesis of *Salmonella* in host cells, a topic explored in few studies, remains unknown. In *Escherichia coli, yqiC* is involved in inducing oxidative stress response in host cell membranes (Al Mamun et al., 2012). We previously used Vector NTI v10 (Invitrogen, Carlsbad, CA, USA) to compare the amino acid sequences of the YqiC of *Salmonella* to those of IbpA and IbpB of *E. coli* (Matuszewska et al., 2008), which exhibited approximately 25% homology. IbpA and IbpB in *E. coli* have been reported to mediate resistance to oxidative stress (Kitagawa et al., 2000). Thus far, 18 putative membrane proteins of the Yqi family have been identified in *Bacillus subtilis* (Kunst et al., 1997). Among these, YqiD and YqiE are possibly involved in the biosynthesis of menaquinone, an electron transport chain mediator (Julsing et al., 2007). Within bacteria, menaquinone serves as an antioxidant (Nowicka and Kruk, 2010), and the electron transport chain is involved in *E. coli* flagellation (Hertz and Bar-Tana, 1977). Therefore, menaquinone can be potentially linked to flagellar biosynthesis for bacterial virulence. Thus, YqiC may be involved in the regulation of menaquinone and virulence in *Salmonella*.

Type-1 fimbrial genes (*fim*) are involved in *Salmonella* colonization in host cells and in the regulation of flagellin. When *Salmonella* reach the intestinal tract, the proteinaceous hair-like structures named fimbriae mediate bacterial adherence to host cells (Duguid and Campbell, 1969). Notably, type-1 fimbriae have a mannose-specific binding capacity, which is responsible for the bacteria–IEC association (Lindquist et al., 1987). The inhibition of *Salmonella* type-1 fimbrial gene expression can attenuate bacterial virulence in swine and mice (Aslanzadeh and Paulissen, 1990; Althouse et al., 2003). The activation of *Salmonella* type-1 fimbriae is regulated by FimZ, a member of the *fim* gene cluster (Yeh et al., 1995). The synthesis of flagella and fimbriae are oppositely controlled because increased expression of FimZ results in non-motility of hyperfimbriated *S*. Typhimurium downregulates the *flhDC* master flagellar operon (Clegg and Hughes, 2002). Flagella are essential for bacterial motility and dissemination during *Salmonella* infection (Duan et al., 2013). In addition, flagellin is the major and the most extensively studied flagellar protein in the pathogen-associated molecular pattern of *Salmonella*, and is also a key virulence factor in bacterial invasion and host response induction (Kawai and Akira, 2011). Salmonellosis is characterized by the recruitment of neutrophils from the microvasculature to the infected intestines (Cheminay et al., 2004), driven by IL-8 released from the IECs (McCormick et al., 1993) after *Salmonella* flagellin binds with toll-like receptor 5 of IECs, subsequently activating the cellular nuclear factor NF-κB and MAPK signaling pathways (Yu et al., 2003; Kawai and Akira, 2011). However, the association between flagella and the non-SPI *yqiC* in *Salmonella* has not been explored.

Defensins affecting the host innate immunity after *Salmonella* infection are small cationic AMPs that have a key role in host defense properties and are present in granulocytic leucocytes, mucosal surface, skin, and other epithelia (Selsted and Ouellette, 2005). AMP expression in the gastrointestinal tract is either constitutive or inducible. hBD-1 is the major constitutive AMP synthesized on the intestinal epithelial surface, whereas hBD-2, hBD-3, and a small amount of hBD-4 are the major inducible proteins expressed only during infection or inflammation (Wehkamp et al., 2007). In *Salmonella*-infected Caco-2 cells, hBD-1 and hBD-2 mRNA expression can be activated (O’Neil et al., 1999), and *Salmonella* flagellin is the primary ligand that induces hBD-2 expression through NF-κB activation (Ogushi et al., 2001; Takahashi et al., 2001). Although, host responses of AvBD-3 and gallinacin-3 can be enhanced in the gastrointestinal tissues of broiler chickens after *Salmonella* infection (Ramasamy et al., 2012), whether hBD-3 expression is affected by *Salmonella* infection in the human intestinal epithelium remains unknown.

To investigate the effects of *yqiC* on *Salmonella*–host interaction, we determined whether *yqiC* affects the expression of virulence factors in *Salmonella* and the IL-8- and hBD-3-related innate immune responses of the human intestinal epithelial LS174T cells. According to the findings of morphological studies, we performed yeast agglutination tests and qRT-PCR analyses to confirm our hypothesis that *yqiC* expression upregulates type-1 fimbrial expression and downregulates flagellar, SPI-1, and SPI-2 gene expression. We also reviewed relevant literature to determine the correlation between *yqiC* and the electron transport chain and performed two chromatography techniques to examine whether *yqiC* affects menaquinone expression in *S*. Typhimurium. Rotenone inhibition assays were performed to determine whether the effect of the NADH dehydrogenase inhibitor in the electron transport chain was similar to that of *yqiC* in regulating selected type-1 fimbrial, flagellar, SPI-1, and SPI-2 gene expression in *S*. Typhimurium.

## Materials and Methods

### Bacterial Strains and Culture Conditions

All bacterial strains—the wild-type *S*. Typhimurium strain SL1344 and its isogenic *yqiC*-deleted mutant strain (Δ*yqiC*), *yqiC*-complement Δ*yqiC* strain (Δ*yqiC′*), *fliC*-deleted mutant strain (Δ*fliC*), and *spaS*-deleted SPI-1 mutant strain (Δ*spaS*) were used in this study. SL1344 and Δ*spaS* were kindly provided by Duncan Maskell. *S*. Typhimurium Δ*yqiC* and Δ*fliC* were created using a previously described lambda (λ)-red recombinase method, with slight modifications (Gust et al., 2003). The kanamycin resistance gene cassette containing the *Salmonella yqiC*-flanking sequence was amplified using *yqiC*-specific primers (forward, 5′-AATAAGAGCTAACACTTACCAGTTCAGGGAAACCACAATGAACCGGAATTGCCAGCTG-3′, and reverse, 5′-ATCTTATATGAGCGGGCCGTCAGGCCCGTTCACGTTTTTATCAGAAGAACTCGTCAAG-3′) from the PCRII-TOPO vector. The apramycin resistance gene cassette with *Salmonella fliC*-flanking sequence was amplified using *fliC*-specific primers (forward, 5′-CCAATAACATCAAGTTGTAATTGATAAGGAAAAGATCATGATTCCGGGGATCCGTCGAC-3′, and reverse, 5′-TGATTGTGTACCACGTGTCGGTGAATCAATCGCCGGATTATGTAGGCTGGAGCTGCTTC-3′) from the pIJ773 vector. These chimeric sequences were transformed into *S*. Typhimurium SL1344, which were complemented with the pKD20 plasmid. Recombination was performed using the λ-red recombinase method, and the synthesized strains were cultured and selected on Luria-Bertani (LB) agar supplemented with the appropriate antibiotic (50 μg/mL kanamycin or apramycin). For generating the *yqiC*-complemented *S*. Typhimurium strain (Δ*yqiC*′), the *yqiC*-coding sequence was amplified using *yqiC*-specific primers (forward, 5′-GCCTGCTGGCAGTAAAGC-3′, and reverse, 5′-GCTTCCGCTTGCGATTGC-3′) and cloned into the pACYC184 vector for restoring *yqiC* expression. *S*. Typhimurium Δ*yqiC*′ was maintained in LB broth supplemented with 20 μg/mL chloramphenicol at 37°C.

For maintenance, bacteria were cultured in LB broth (Difco/Becton Dickinson) or on LB agar plates at 37°C with appropriate antibiotics, if necessary. For gentamicin protection assay and ELISA (for estimating *in vitro* IL-8 and hBD-3 secretion), mid-log cultures of the *S.* Typhimurium strains were incubated in LB broth by shaking at 225 rpm and 37°C for 3 h when their OD_600_ was approximately 0.8. For TLC and HPLC, *S.* Typhimurium strains were cultured in overnight cultures by shaking at 225 rpm and 37°C. For other assays, the overnight cultures of the *S.* Typhimurium strains were prepared by incubating single bacterial colonies from LB agar plates into LB broths and then grown at 37°C for approximately 18 h.

### Cell Culture and Bacterial Infections

The human colon carcinoma cell line LS174T [American Tissue Culture Collection (ATCC), Rockville, MD, USA; ATCC-CL188], the colon carcinoma cell line Caco-2 (ATCC-TB37), the human cervical adenocarcinoma cell line HeLa (ATCC-CCL2), and the human monocyte cell line THP-1 (ATCC-TIB202) were cultured according to the ATCC instructions and maintained at 5% CO_2_ and 37°C. When the cells attained 90% confluency, the adherent cells were subcultured through trypsinization by using a trypsin-EDTA solution (Gibco).

For bacterial colonization and invasion assays in triplicate, 5 × 10^5^ LS174T, Caco-2, HeLa, or THP-1 cells were seeded per well onto 12-well plates and cultured for 4–5 days (final cell concentration, approximately 2 × 10^6^ cells/well). Only THP-1 cells were treated with 10 ng/mL phorbol 12-myristate 13-acetate (Sigma-Aldrich) for cell attachment in wells before infection assays. The media of all four cell lines were replaced with FBS-free media before bacterial inoculation, and the cells were then infected with mid-log cultures of *S.* Typhimurium SL1344, Δ*yqiC*, and Δ*yqiC*′ (MOI = 5, i.e., approximately 1 × 10^7^ CFU/well) for 2 h. Subsequently, the infected cells were washed with PBS three times to eliminate non-adherent non-invading bacteria, incubated in FBS-free plain media without gentamicin for 1 h, washed with PBS three times, and lysed using 1% Triton X-100 (Sigma-Aldrich) to generate an output pool A containing cell-associated bacteria. After the initial 2-h infection, the infected cells in the remaining wells were incubated in FBS-free media supplemented with 100 μg/mL gentamicin (Sigma-Aldrich) for 1 h to kill extracellular bacteria. Then, these cells were washed with PBS three times and lysed using 1% Triton X-100 to generate the output pool B containing intracellular bacteria. Serial dilutions of the bacterial lysates from output pools A and B were plated onto LB agar plates, and the CFUs were counted after overnight incubation to determine the number of viable bacteria in the CFUs in terms of the initial inoculum, expressed as CFU per initial inoculum of 2.5 × 10^6^ cells.

### Enzyme-Linked Immunosorbent Assay

For quantifying protein secretion from host cells after infection or stimulation, LS174T cells in individual wells were treated with LB broth containing *S.* Typhimurium SL1344, Δ*yqiC*, Δ*yqiC′*, or Δ*fliC* (MOI = 5); 50 ng/mL IL-1β (a proinflammatory cytokine; R&D System Europe); or 100 ng/mL *Salmonella* flagellin (InvivoGen) for 2 h by using the same protocol as earlier prescribed in the bacterial invasion assay to generate output pool B. Next, they were initially incubated in media supplemented with 100 μg/mL gentamicin for 1 h and then in media supplemented with 10 μg/mL gentamicin for an additional 15 h. After the above 18-h treatment, the media from the final 15-h incubation in these wells were harvested and centrifuged, and the supernatants were collected for performing ELISAs for IL-8 and hBD-3 in triplicate according to manufacturer’s instructions (human IL-8 ELISA development kit and human BD-3 ELISA development kit; PreproTech EC). IL-8 and hBD-3 concentrations in the media were determined at 405 nm on a SpectraMax reader (Molecular Devices, Sunnyvale, CA, USA) and calculated according to the standard curves plotted from serially diluted standard solutions using Microsoft Office Excel 2007.

### Transmission Electron Microscopy

For morphological evaluation of *S*. Typhimurium strains through TEM, a loopful of bacteria was obtained from the colonies of each strain grown on LB agar plates and suspended in 300 μL of PBS. Resuspended bacterial solutions were centrifuged and washed with PBS two times. After another round of centrifugation, the supernatants were removed, and the bacterial pellets were then negatively stained using 2% phosphotungstic acid (Sigma-Aldrich). Finally, the stained bacterial suspensions were deposited on a carbon-coated grid, and all samples were observed under a Hitachi H-600 transmission electron microscope (Hitachi, Ltd, Tokyo, Japan).

### Yeast Agglutination Test

Yeast agglutination tests were performed in triplicate as described previously (Wang et al., 2012). Each bacterial strain was streaked on LB agar plates and then incubated at 28 and 37°C for 18 h. A loopful of bacterial colonies was obtained from the LB agar plates and resuspended in 100 μL of PBS. Next, 30 μL of bacterial suspension was mixed with an equal amount of 3% (w/v) *Saccharomyces cerevisiae* cells (Sigma-Aldrich) on a glass slide. The formation of agglutination fragments indicated mannose-sensitive fimbriae adherence. In addition, the bacterial suspension was mixed with 3% (w/v) *S. cerevisiae* cells and additional 3% (w/v) D-mannose solution (Sigma-Aldrich) for confirming whether yeast agglutination was caused by mannose-specific binding mediated by type-1 fimbriae.

### Biofilm Formation Assay

Biofilm formation assays were performed in triplicate as described previously (O’Toole, 2011). The overnight cultures of *S.* Typhimurium SL1344, Δ*yqiC*, and Δ*yqiC′* were diluted in LB broth without salt, and 100 μL of each bacterial dilution was added into each well in triplicate in 96-well microplates for additional incubation at 37°C for 18 h. After incubation, the bacteria were removed and washed with PBS. Then, 125 μL of 0.1% crystal violet (Sigma-Aldrich) was added into each well, which was subsequently washed with PBS. Finally, 125 μL of 30% acetic acid (Sigma-Aldrich) was added into each well. The absorbance in each well was determined at 550 nm on the SpectraMax reader (Molecular Devices).

### Bacterial Motility Assay

The bacterial motility assays were performed in triplicate. Semisolid LB agar plates containing 0.3% agar were used for the bacterial motility assay. Overnight-cultured bacteria (10 μL) were inoculated onto the centers of the semisolid LB agar plates, which were incubated at 37°C for 6 h. The diameters of the growth zones represent the strength of bacterial motility. *S*. Typhimurium Δ*fliC* and Δ*spaS* were used as the negative and positive controls, respectively.

### qRT-PCR Analysis

Total RNA of *S*. Typhimurium SL1344, Δ*yqiC*, and Δ*yqiC′* was isolated from their overnight cultures by using the Total RNA Miniprep Purification Kit (Genemark, Taichung, Taiwan) according to manufacturer’s protocol. Total RNA samples were cleaned and purified using RNase-free DNase I (1 unit/1 μg RNA; NEB, Beverly, MA, USA). Next, 0.5 μg of RNA was reverse transcribed to cDNA using the Transcriptor High Fidelity cDNA Synthesis Kit (Roche Applied Science, Mannheim, Germany) according to the manufacturer’s instruction. Oligonucleotide primer pairs specific to the target genes, namely *fimA, fimZ, flhD, fliZ, invF*, and *sseB*, and the housekeeping 16S ribosomal RNA gene (**Table 1**) were designed using Primer3 and BLAST^1^. By using the Bio-Rad C100 Real-Time PCR System, 0.1 μg of cDNA was amplified in a 20-μL reaction solution containing 0.25 μM of each primer and 10 μL of iQ SyBr green supermix (BioRad) with 40 cycles of enzyme activation at 95°C for 3 min, denaturation at 95°C for 15 s, annealing at 48°C for 30 s, and extension at 72°C for 30 s. The mRNA transcription levels were calculated using the ^ΔΔ^Ct method as described previously (Livak and Schmittgen, 2001), and the expression levels of 16S ribosomal RNA were used for normalization. The mRNA expression levels of *fimA, fimZ, flhD, fliZ, invF*, and *sseB* were expressed as fold-change relative to *S*. Typhimurium SL1344.

**Table 1:** Primers used for qRT-PCR.

Primer (F: forward, R: reverse)	Sequence (5′–3′)	Product size (base pairs)	Description
*fimA*-F	CTAAATCCGCCGATCAAA	193	Type-1 fimbrial gene
*fimA*-R	GAGGAGACAGCCAGCAAA	193	Type-1 fimbrial gene
*fimZ*-F	GACGAACACCCTATTGTAAGA	202	Type-1 fimbrial gene
*fimZ*-R	GTTCCTGGATAGATTTGATTC	202	Type-1 fimbrial gene
*flhD*-F	GCATACATCCGAGTTGCTAA	237	Flagellar gene
*flhD*-R	TGAGTCAAACGGGTGATC	237	Flagellar gene
*fliZ*-F	GGAATATGTCGTGCGTTTA	221	Flagellar gene
*fliZ*-R	TATCAGAACTGGCGGTAAA	221	Flagellar gene
*invF*-F	TGAGAATGCTGGGAGAAGAC	194	SPI-1 gene
*invF*-R	AAAATGTGAAGGCGATGAGT	194	SPI-1 gene
*sseB*-F	AACGTGCCAGAAATACCCAG	199	SPI-2 gene
*sseB*-R	CCTTTATCCAGCTTCCCATG	199	SPI-2 gene
16S-F	TTCCTCCAGATCTCTACGCA	552	Housekeeping gene 16S
16S-R	GTGGCTAATACCGCATAACG	552	Housekeeping gene 16S

### NADH Dehydrogenase Inhibition Assay

To determine the involvement of the electron transport chain in *Salmonella yqiC* virulence, 100 μg/mL rotenone (Sigma-Aldrich), an NADH dehydrogenase inhibitor (Ueno et al., 1994), was added to the *S.* Typhimurium SL1344 cultures in LB broth and incubated at 37°C for 18 h. The total RNA of the prepared overnight cultures of *S*. Typhimurium SL1344 and Δ*yqiC* and *S*. Typhimurium SL1344 mixed with rotenone were isolated and processed in triplicate as described for the qRT-PCR analysis. The mRNA expression levels of *fimA, fimZ, flhD, fliZ, invF*, and *sseB* in *S*. Typhimurium SL1344 mixed with rotenone were compared with those in *S*. Typhimurium Δ*yqiC*.

### Menaquinone Complementation Assay

To study the role of menaquinone in *Salmonella yqiC* virulence, 100 μg/mL menaquinone (Sigma-Aldrich) was added into the LB broth containing *S*. Typhimurium Δ*yqiC* and then coincubated at 37°C for 18 h. The total RNA of *S*. Typhimurium Δ*yqiC* was isolated and processed as described for the qRT-PCR analysis. The mRNA expression levels of *fimA, fimZ, flhD, fliZ, invF*, and *sseB* in the *S*. Typhimurium Δ*yqiC* complemented with menaquinone were compared with those in *S*. Typhimurium SL1344.

### TLC for Menaquinone

We extracted menaquinone from *S*. Typhimurium SL1344, Δ*yqiC*, and Δ*yqiC′* according to a protocol described previously (Kwon et al., 2000). All bacterial strains were cultured in 500 mL of 0.5% glucose and minimal salt medium (33.9 g/L Na_2_HPO_4_.7H_2_O, 15 g/L KH_2_PO_4_, 5 g/L NH_4_Cl, and 2.5 g/L NaCl) with vigorous shaking and incubated at 37°C overnight. The bacteria were harvested by centrifugation at 10,000 rpm, and the supernatants were discarded. The pellets were suspended in acetone and filtered through Whatman No. 1 filter papers (pore size, 11 μm; thickness, 0.18 mm). The filtrates were dried under reduced pressure. Two drops of acetone were added to the dried materials for resuspension. Finally, the suspensions were spotted on a Baker Si250F silica gel plate (J. T. Baker, Phillipsburg, NJ, USA) and separated using petroleum ether–ethyl ether (70:30) or chloroform.

### HPLC for Menaquinone

For reconfirming the presence of menaquinone in *S*. Typhimurium SL1344, Δ*yqiC*, and Δ*yqiC′*, the bacterial menaquinone was extracted using acetone, as described in the TLC procedure. The acetone extracts were centrifuged and filtered using Whatman No. 1 filter papers to remove cell debris. Subsequently, the filtered extracts were dried under decreasing pressure and treated with 1 mL of acetonitrile. The reconstituted mixtures were filtered using Sep-Pak C18 cartridges (Waters, Milford, MA, USA) and subjected to HPLC (Hitachi L-7400) on a Syncronis C18 HPLC column (Thermo Fisher Scientific, Waltham, MA, USA). Menaquinone was detected at 245 nm on a UV detector.

### Statistical Analysis

All experiments were performed in triplicate, and data were analyzed using the Student’s *t*-test to compare the differences between the control and *S*. Typhimurium groups. A *p*-value of <0.05 was considered statistically significant.

## Results

### *yqiC* Deletion Inhibits *S*. Typhimurium Colonization and Invasion of Four Human Cell Lines

**FIGURE 1:**
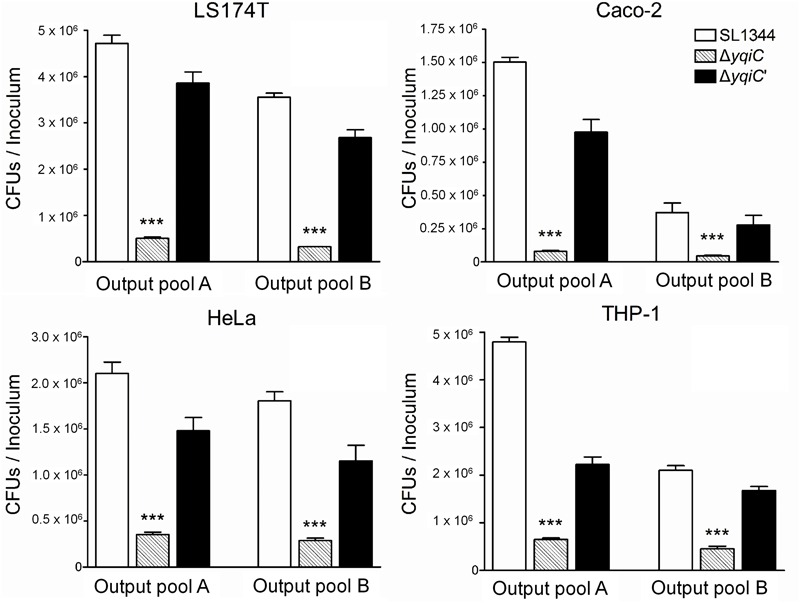
**Colonization and invasion rates of three *Salmonella* Typhimurium strains in four human cell lines.** LS174T, Caco-2, HeLa, and THP-1 cells were infected with *S.* Typhimurium SL1344, Δ*yqiC*, and Δ*yqiC′*. The CFUs in output pools A and B represent the colonized and intracellular bacteria, respectively. The CFUs of the cell lysates containing bacteria were counted, and the bacterial counts of the colonizing and invading *S.* Typhimurium Δ*yqiC* and Δ*yqiC′* CFUs were compared with those of the colonizing and invading *S.* Typhimurium SL1344 using the Student’s *t*-test. ^∗∗∗^*p* < 0.001.

To investigate whether *yqiC* affects the colonizing and invading abilities of *S*. Typhimurium, three non-phagocytic human epithelial cell lines and one phagocytic cell line were infected with *S*. Typhimurium SL1344, Δ*yqiC*, and Δ*yqiC′*. Our bacterial colonization and invasion assays demonstrated that approximately 4.8 × 10^6^, 2.2 × 10^6^, and 1.5 × 10^6^ CFU of *S*. Typhimurium SL1344 cells colonized LS174T and THP-1 (at similar levels), HeLa, and Caco-2 cells, respectively (white bars in output pools A, **Figure 1**). The internalization rates of *S*. Typhimurium SL1344 were the highest in LS174T cells, followed by THP-1, HeLa, and Caco-2 cells (white bars in output pools B, **Figure 1**). Deletion of *yqiC* significantly inhibited the colonization and invasion of *S*. Typhimurium in all four cell types (striated bars, **Figure 1**). Furthermore, 20–50% inhibition of *S*. Typhimurium Δ*yqiC* in colonization and invasion was partially recovered by the complementation of *yqiC* in the output pool A or B (black bars, **Figure 1**), thus suggesting the significance of *yqiC* to the colonizing and invading abilities of *S*. Typhimurium. Therefore, *yqiC* is required in the early pathogenesis *S*. Typhimurium, thus clarifying its significance to the colonization and invasion of *S*. Typhimurium in various host cells, irrespective of the cell type.

### *yqiC* and *fliC* Contribute to IL-8 Secretion of LS174T Cells after *S*. Typhimurium Infection

**FIGURE 2:**
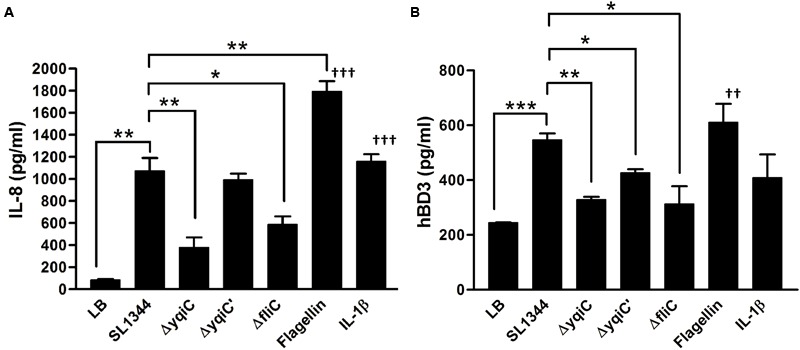
**Secretion of IL-8 and hBD-3 in LS174T cells after treatment with *S.* Typhimurium SL1344, Δ*yqiC*, Δ*yqiC′*, Δ*fliC, Salmonella* flagellin, and IL-1β.** LS174T cells were treated with *S.* Typhimurium SL1344 (MOI = 5), 100 ng/mL *Salmonella* flagellin, and 50 ng/mL IL-1β, according to the protocol in the bacterial invasion assay to generate output pool B. After 18-h treatment, the media from the final 15-h incubation in these wells were examined for quantification of IL-8 **(A)** and hBD-3 **(B)** secretion by using ELISA. Flagellin- and IL-1β-treated cells were the positive controls. The Student’s *t*-test was used for analyzing any significant differences in IL-8 and hBD-3 secretion levels between the *S*. Typhimurium SL1344-infected cells and the other groups (^∗^*p* < 0.05, ^∗∗^*p* < 0.01, ^∗∗∗^*p* < 0.001), and significant differences in IL-8 and hBD-3 secretion levels between negative control (LB-treated cells) and positive controls (^††^*p* < 0.01, ^†††^*p* < 0.001).

To determine whether *Salmonella yqiC* affects the induction of host inflammation, confluent LS174T cells were treated with *S*. Typhimurium SL1344, Δ*yqiC*, Δ*yqiC′*, and Δ*fliC*; IL-1β; and *Salmonella* flagellin for examining host IL-8 secretion. IL-8 secretion was significantly augmented in the LS174T cells treated with IL-1β and *S*. Typhimurium SL1344 compared with that in untreated cells (**Figure 2A**). Moreover, the secretion was higher in the cells treated with *Salmonella* flagellin than in those treated with IL-1β. In addition, IL-8 secretion significantly decreased in the LS174T cells infected with *S*. Typhimurium Δ*yqiC* and Δ*fliC* compared with those infected with *S*. Typhimurium SL1344. The IL-8 secretion levels in the *S*. Typhimurium Δ*yqiC′*- and SL1344-infected cells were similar (**Figure 2A**). Two positive controls, *Salmonella* flagellin and IL-1β (at the administered dosage) significantly increased IL-8 production compared with untreated controls (**Figure 2A**). Thus, disruption of either *yqiC* or *fliC* remarkably suppressed *S*. Typhimurium-induced IL-8 production in LS174T cells. Thus, *Salmonella* flagellin and YqiC have a crucial role in the activation of IL-8 release from LS174T cells after *S*. Typhimurium infection.

### *yqiC* and *fliC* Are Required for hBD-3 Production in LS174T Cells after *S*. Typhimurium Infection

Because hBD-3 can be expressed in LS174T cells (Fahlgren et al., 2004), but not in Caco-2 cells (Ou et al., 2009), we selected LS174T cells as an *in vitro* model for studying the involvement of *yqiC* and *fliC* in hBD-3-related host innate immunity in human IECs. Confluent LS174T cells were treated with *S*. Typhimurium SL1344, Δ*yqiC*, Δ*yqiC′*, and Δ*fliC*; IL-1β; and *Salmonella* flagellin for determining host hBD-3 production. *S*. Typhimurium significantly induced increased hBD-3 production in LS174T cells (**Figure 2B**). By contrast, hBD-3 secretion significantly decreased in the LS174T cells infected with *S*. Typhimurium Δ*yqiC* and Δ*fliC* compared with those infected with *S*. Typhimurium SL1344, and Δ*yqiC′* partially restored hBD-3 secretion levels (**Figure 2B**). Thus, *yqiC* and *fliC* have a similar effect on hBD-3 production in LS174T cells after *S*. Typhimurium infection. *Salmonella* flagellin, but not IL-1β (at the administered dosage), significantly increased hBD-3 production compared with untreated controls (**Figure 2B**). The proinflammatory cytokine IL-1β appeared to be an inefficient positive control compared with *Salmonella* flagellin in terms of hBD-3 secretion in LS174T cells. Therefore, LS174T cells produce hBD-3 after *S*. Typhimurium infection, and *yqiC* and *fliC* of *S*. Typhimurium contribute to host hBD-3 expression. Thus, we hypothesized that *yqiC* of *S*. Typhimurium affects flagellation, flagella-related functions, and host responses.

### TEM Revealed Fewer and Shorter Flagella and Presence of Fimbriae in *S*. Typhimurium Δ*yqiC*

**FIGURE 3:**
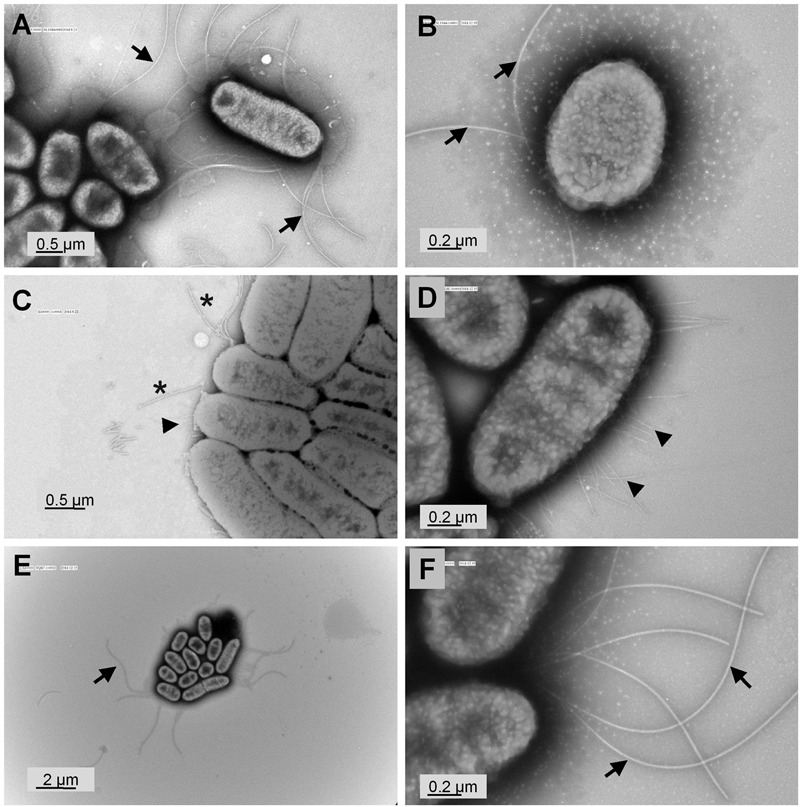
**Transmission electron micrographs of *S*. Typhimurium SL1344 and Δ*yqiC*.** Representative transmission electron micrographs after negative staining of at least four different fields of view from each bacterial strain reveal the morphology of *S*. Typhimurium SL1344 **(A,B)**, Δ*yqiC* **(C,D)**, and Δ*yqiC′* **(E,F)**. Numerous long flagella (arrows) were observed on the surfaces of *S*. Typhimurium SL1344 [magnification: **(A)** 30,000×; **(B)** 80,000×]. Type-1 fimbriae (arrowheads) and defective flagella (asterisks) were observed in *S*. Typhimurium Δ*yqiC* [magnification: **(C)** 40,000×; **(D)** 80,000×]. Similar to *S*. Typhimurium SL1344, numerous long flagella without fimbriae were observed in *S*. Typhimurium Δ*yqiC′* [magnification: **(E)** 10,000×; **(F)**: 80,000×].

To validate our hypothesis that *yqiC* is responsible for *S*. Typhimurium flagellation, cell surfaces of *S*. Typhimurium SL1344, Δ*yqiC*, and Δ*yqiC′* were examined through TEM after negative staining. The results revealed remarkable differences in the bacterial surfaces of the wild-type and mutant *S.* Typhimurium strains. *S.* Typhimurium SL1344 exhibited numerous intact long flagella surrounding the bacterial cell (**Figures 3A,B**). By contrast, only a few of the fragmented flagella with short shafts surrounding the bacterial cells were observed in *S.* Typhimurium Δ*yqiC*, with identifiable type-1 fimbriae-like appendages on their cell surfaces (**Figures 3C,D**). *S.* Typhimurium Δ*yqiC′* exhibited identical flagellar expression and the absence of fimbriae-mimicking structures, as observed in *S.* Typhimurium SL1344 (**Figures 3E,F**). Thus, *yqiC* deletion impairs flagella formation and activates the expression of type-1 fimbriae-like structures on the bacterial cell surface.

### *yqiC* Deletion Enhances Type-1 Fimbrial Expression in *S*. Typhimurium

**FIGURE 4:**
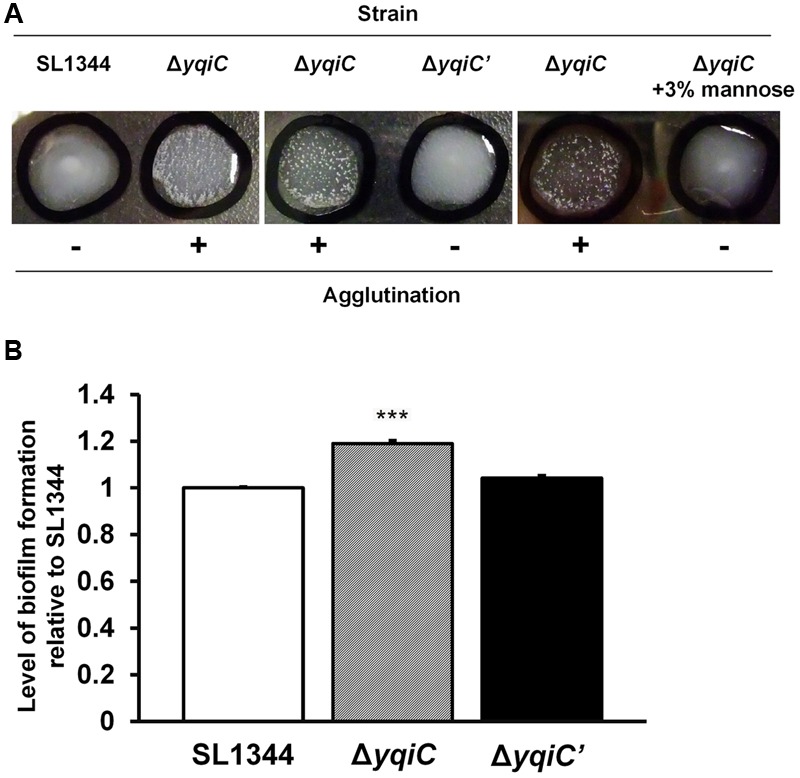
**Phenotypic analysis of type-1 fimbrial expression in *S*. Typhimurium SL1344, Δ*yqiC*, and Δ*yqiC′.* (A)** Yeast agglutination tests for *S*. Typhimurium strains incubated at 37°C. Formation of small agglutination fragments in *S*. Typhimurium SL1344 Δ*yqiC* indicated that the bacteria can express type-1 fimbriae and agglutinate with the yeast cells. The addition of 3% D-mannose interfered with receptor binding on the surface of *S*. Typhimurium SL1344 and Δ*yqiC*, which could not agglutinate with yeast cells to form small fragments, thus confirming that the *yqiC* deletion activated the expression of type-1 fimbriae. **(B)** The biofilm formation assay. *S*. Typhimurium SL1344, Δ*yqiC* and Δ*yqiC′* were cultured in 96-well microplates at 37°C for 18 h. Then, all wells were washed with PBS and stained using 0.1% crystal violet for subsequent measurement of the absorbance detected at OD_550_. The relative levels of biofilm formation in *S*. Typhimurium Δ*yqiC* and Δ*yqiC′* were compared with those in *S*. Typhimurium SL1344 using the Student’s *t*-test. ^∗∗∗^*p* < 0.001.

To investigate whether *yqiC* deletion activates type-1 fimbrial expression, we performed yeast agglutination tests for determining the mannose-specific binding of type-1 fimbriae in *S.* Typhimurium SL1344, Δ*yqiC*, and Δ*yqiC′*. No agglutination was observed in *S.* Typhimurium SL1344. However, significant yeast agglutination was observed in *S.* Typhimurium Δ*yqiC*, and small fragments disappeared in *S.* Typhimurium Δ*yqiC′*, respectively (left and middle panels, **Figure 4A**). Nonetheless, when D-mannose was added to the bacterial suspension of *S.* Typhimurium Δ*yqiC* to block the binding of its type-1 fimbriae with yeast, these fragments disappeared and this phenomenon appeared similar to the result in *S.* Typhimurium SL1344 (right, **Figure 4A**). Therefore, *yqiC* can suppress mannose-specific type-1 fimbrial expression in *S*. Typhimurium. Further, the biofilm formation assays demonstrated that that the biofilm formation was enhanced in *S.* Typhimurium Δ*yqiC* (**Figure 4B**), and it was restored in *S.* Typhimurium Δ*yqiC′*. According to these results, *yqiC* can prevent biofilm formation by inhibiting type-1 fimbrial expression in *S*. Typhimurium.

### *yqiC* Is Involved in *S*. Typhimurium Motility

**FIGURE 5:**
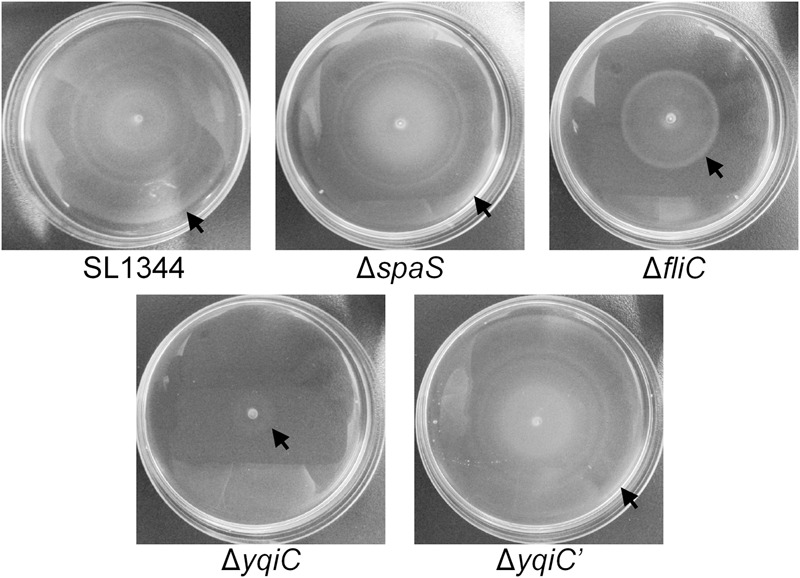
**Bacterial motility assays for *S*. Typhimurium SL1344, Δ*yqiC*, Δ*yqiC′*, Δ*fliC*, and Δ*spaS*.** The motilities of *S*. Typhimurium SL1344, Δ*yqiC*, Δ*yqiC′*, Δ*fliC*, and Δ*spaS* were examined through bacterial inoculation on semisolid LB agar plates, followed by a 6-h incubation at 37°C. The arrows indicate the outer rims of the motility zones.

*yqiC* is required for *S*. Typhimurium colonization and invasion and for inducing *fliC*-like IL-8 and hBD-3 secretion in host cells. Moreover, *fliC* is involved in bacterial motility. To examine whether *yqiC* contributes to bacterial motility, we compared the maximal diameters of the motility zones of five *S*. Typhimurium strains and observed that the diameter of the motility zone of the positive control *S*. Typhimurium Δ*fliC* was smaller than that of *S*. Typhimurium SL1344, whereas the diameters of the motility zones of *S*. Typhimurium SL1344 and its SPI-1 mutant Δ*spaS* were equal (upper, **Figure 5**). Moreover, the diameter of the motility zone of *S*. Typhimurium Δ*yqiC* was smaller than that of *S*. Typhimurium SL1344 and Δ*fliC* (**Figure 5**). The diameters of the motility zone of *S*. Typhimurium SL1344 was almost equal to that of *S*. Typhimurium Δ*yqiC′* (**Figure 5**). Therefore, the loss of *yqiC* can downregulate *S*. Typhimurium motility, and its effect is more prominent than that of the loss of *fliC*.

### *yqiC* Deletion Upregulates *fim* Gene Expression and Downregulates Flagella, SPI-1, and SPI-2 Gene Expression in *S*. Typhimurium

**FIGURE 6:**
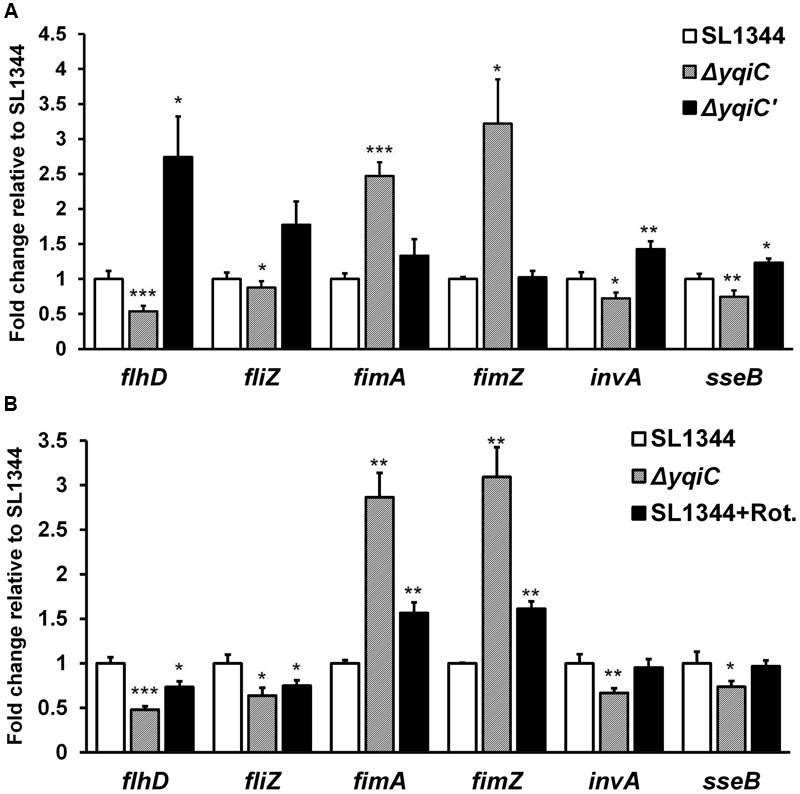
**Gene expression analysis of flagella, type-1 fimbriae, SPI-1, and SPI-2 in *S*. Typhimurium SL1344, Δ*yqiC*, and Δ*yqiC′*, and NADH dehydrogenase inhibition assays using qRT-PCR. (A)** The mRNA expression levels of the type-1 fimbriae-related genes *fimA* and *fimZ*, the flagella-related genes *flhD* and *fliZ*, the SPI-1-related gene *invA*, and the SPI-2 gene *sseB* in *S*. Typhimurium Δ*yqiC* were compared with those of *S*. Typhimurium SL1344 and Δ*yqiC′*. **(B)** The NADH dehydrogenase inhibition assay revealed the effects of rotenone (Rot) and *yqiC* deletion on the regulation of the mRNA expression in *flhD, fliZ, fimA*, and *fimZ, invA* and *sseB*. The mRNA expression levels of the target genes are expressed as fold-change relative to the geometric means of their mRNA expression levels in *S*. Typhimurium SL1344. Any significant differences in the mRNA expression levels between *S*. Typhimurium SL1344 and other strains or conditions were analyzed using the Student’s *t*-test. ^∗^*p* < 0.05, ^∗∗^*p* < 0.01, and ^∗∗∗^*p* < 0.001 were considered statistically significant.

To further delineate the role of *yqiC* in regulating type-1 fimbrial, flagellar, SPI-1, and SPI-2 gene expression, we performed qRT-PCR for *S*. Typhimurium SL1344, Δ*yqiC*, and Δ*yqiC′*. Compared with their expression in *S*. Typhimurium SL1344, *fimA* and *fimZ* expression was significantly upregulated in *S*. Typhimurium Δ*yqiC* (2.5–3 fold-change, *p* < 0.05), whereas their expression in *S*. Typhimurium Δ*yqiC′* was similar to that in *S*. Typhimurium SL1344 (**Figure 6A**). By contrast, *flhD, fliZ, invA*, and *sseB* expression was significantly downregulated in *S*. Typhimurium Δ*yqiC* (-0.5 to -0.2 fold-change, *p* < 0.05; **Figure 6A**). In *S*. Typhimurium Δ*yqiC′*, the mRNA expression of the preceding genes was restored (**Figure 6A**). Our qRT-PCR results were consistent with the *yqiC* phenotypes obtained in TEM, yeast agglutination tests, and gentamicin protection and bacterial motility assays.

### NADH Dehydrogenase Inhibition by Rotenone in the Electron Transport Chain Is Similar to Type-1 Fimbrial, Flagellar, SPI-1, and SPI-2 Gene Regulation in *S*. Typhimurium Δ*yqiC*

To study whether the electron transport chain is involved in regulating the phenotype of *yqiC*, we conducted inhibition assays by using rotenone, an NADH dehydrogenase inhibitor (Ueno et al., 1994), and investigated the effects of the electron transport chain on the *yqiC*-regulated expression of flagellar, type-1 fimbrial, SPI-1, and SPI-2 genes. The qRT-PCR results indicated that *flhD* and *fliZ* were significantly downregulated in rotenone-treated *S*. Typhimurium SL1344 and *S*. Typhimurium Δ*yqiC* (both *p* < 0.05; black and striated bars, respectively, **Figure 6B**). Rotenone significantly upregulated *fimA* and *fimZ* expression in *S*. Typhimurium SL1344 (both *p* < 0.01), although their expression levels were not as high as those in *S*. Typhimurium Δ*yqiC* (black and striated bars, respectively, **Figure 6B**). By contrast, rotenone had no significant effect on *invA* and *sseB* expression, in contrast to the downregulation induced by *yqiC* knockout in *S*. Typhimurium (**Figure 6B**). Therefore, rotenone inhibition resulted in a regulatory effect similar to that of *yqiC* deletion on flagella and type-1 fimbrial expression, suggesting that the electron transport chain is involved in *yqiC* phenotype expression. Whether the electron transport chain is linked to the *yqiC*-related modulation of flagellation and type-1 fimbriae activation in *S*. Typhimurium requires further studies for clarification. However, *yqiC* possibly regulates SPI-1 and SPI-2 gene expression in *S*. Typhimurium through pathways not associated with NADH dehydrogenase in the electron transport chain.

### *yqiC* Is Indispensable for Menaquinone Biosynthesis in *S*. Typhimurium

**FIGURE 7:**
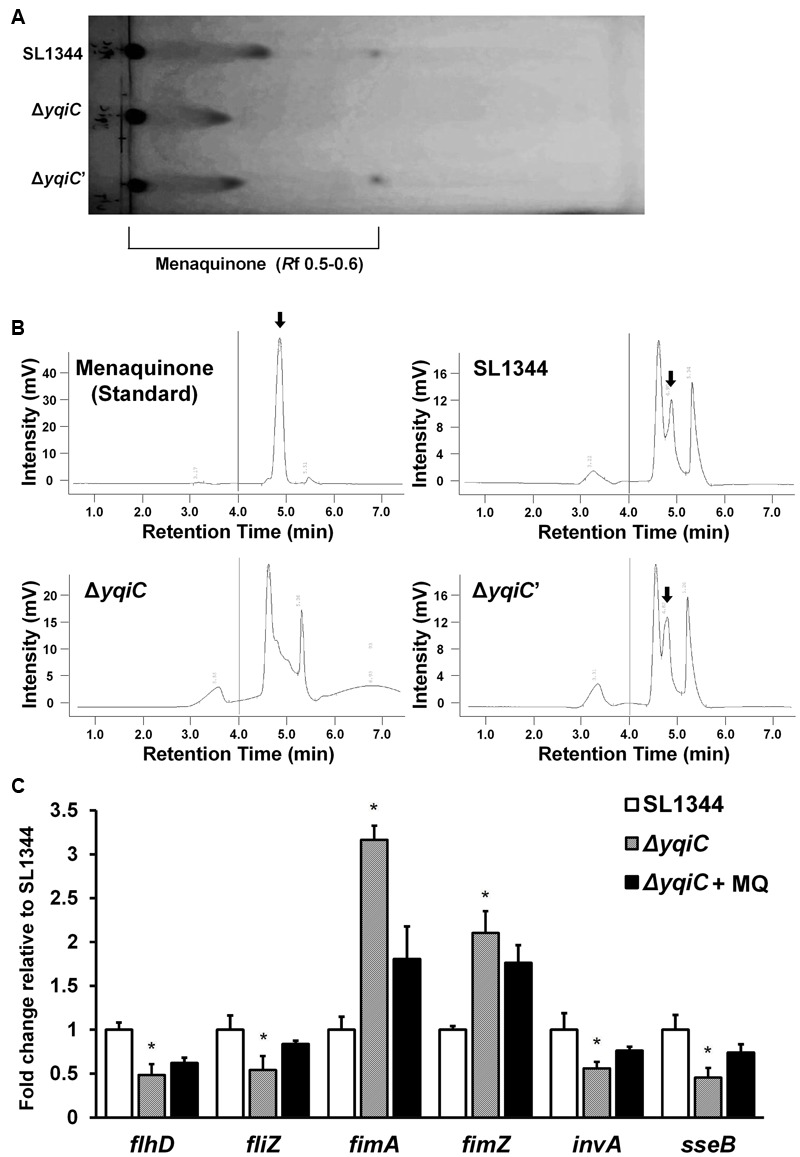
**Thin-layer chromatography and HPLC for menaquinone in *S*. Typhimurium SL1344, Δ*yqiC*, and Δ*yqiC′*, and menaquinone complementation assays using qRT-PCR.** The menaquinone content within *S*. Typhimurium SL1344, Δ*yqiC*, and Δ*yqiC′* was detected using TLC and HPLC. **(A)** TLC revealed the presence of menaquinone at an *R*_f_ of 0.5–0.6 in *S*. Typhimurium SL1344 and Δ*yqiC′*, but detectable menaquinone was absent in *S*. Typhimurium Δ*yqiC*. **(B)** HPLC revealed menaquinone peaks at a retention time of 4.90 min in *S*. Typhimurium SL1344 and Δ*yqiC′* (arrows), but menaquinone peaks were absent in *S*. Typhimurium Δ*yqiC*. **(C)** In the menaquinone complementation assay, menaquinone (MQ) was added into the LB broth containing *S*. Typhimurium Δ*yqiC* for 18-h overnight culture. The mRNA expression levels of *flhD, fliZ, fimA, fimZ, invA*, and *sseB* of *S*. Typhimurium SL1344 Δ*yqiC* and menaquinone-complemented *S*. Typhimurium Δ*yqiC* were compared with those of *S*. Typhimurium SL1344 and are expressed as fold-change relative to the geometric means of their mRNA expression levels in *S*. Typhimurium SL1344. Any significant differences in the mRNA expression levels between *S*. Typhimurium SL1344 and other strains or conditions were analyzed using the Student’s *t*-test. ^∗^*p* < 0.05 was considered statistically significant.

Although, another two *yqi* genes *yqiE* and *yqiD* are involved in menaquinone biosynthesis in *Bacillus subtilis* (Julsing et al., 2007), it remains unknown whether *yqiC* is associated with the biosynthesis of the crucial electron transport chain mediator menaquinone in *S*. Typhimurium. We extracted and identified menaquinone contents from the cell membranes of *S*. Typhimurium SL1344, Δ*yqiC*, and Δ*yqiC′* through TLC and HPLC. The spots eluted farthest from the start line on the TLC plate represented menaquinone (*R*_f_ 0.5-0.6, **Figure 7A**), whereas the secondary spots, also farther from the start line, were considered other quinone-like compounds or menaquinone intermediates (*R*_f_ 0.3-0.4, **Figure 7A**). TLC revealed that menaquinone was present in *S*. Typhimurium SL1344 and Δ*yqiC*′, but not in *S*. Typhimurium Δ*yqiC*. Furthermore, we performed HPLC for the membrane fraction components of the three *S*. Typhimurium strains to reconfirm our TLC results and used the commercial menaquinone K2 as a measurement standard (retention time 4.90 min). In HPLC, menaquinone peaks were only observed for *S*. Typhimurium SL1344 and Δ*yqiC* and not for *S*. Typhimurium Δ*yqiC* (**Figure 7B**).

### Menaquinone Reverses the Effect of *yqiC* Deletion on Expression of Type-1 Fimbrial, Flagellar, SPI-1, and SPI-2 Genes in *S*. Typhimurium

To determine the role of menaquinone in *Salmonella* after *yqiC* deletion, menaquinone was added in *S*. Typhimurium Δ*yqiC* for 18-h incubation. The qRT-PCR results indicated that the downregulated expression of *flhD, fliZ, invA*, and *sseB* as well as the upregulated expression of *fimA* and *fimZ* were minimized after additional menaquinone treatment in *S*. Typhimurium Δ*yqiC* (**Figure 7C**). On simulating *S*. Typhimurium SL1344, complementation of menaquinone in the menaquinone-deficient *S*. Typhimurium Δ*yqiC* strain can recover the mRNA expression of type-1 fimbrial, flagellar, SPI-1, and SPI-2 genes.

## Discussion

The phenotype of *yqiC* in *Salmonella* colonization and invasion of host cells might be controlled by environmental temperatures and bacterial cell-growth phases. The importance of *yqiC* in *Salmonella* virulence has been confirmed in mice in a previous study, in which all mice infected with the *yqiC* mutant survived for 30 days, whereas, all mice infected with the wild-type strain died (Carrica et al., 2011). However, the decisive stage of *yqiC* virulence during *S*. Typhimurium infection remains unknown. Although, the *yqiC* mutant completely lost its systemic virulence in mice, when cultured at 28°C, the mutant could invade murine macrophages and human epithelial HeLa cells for up to 24 h post *in vitro* infection as the wild-type (Carrica et al., 2011), hinting a late effect of *yqiC* on its virulence. By contrast, in our study, the *yqiC* mutant cultivated in the mid-log phase at 37°C lost its bacterial colonization and invasion abilities in the human intestinal epithelial LS174T cells during the first 18 h post-infection. The differences in the results of Carrica et al. (2011) and our study may be because of the bacterial virulence, which is influenced by temperature and cell-growth phases. Therefore, at 28°C, the bacteria did not exhibit the *yqiC* phenotype in bacterial colonization and invasion, but at 37°C, the *yqiC* effect was maximized for these two characteristics. Similarly, flagella formation can be enhanced at a low temperature by regulating *clpP* in *Salmonella* (Knudsen et al., 2014), and the *Salmonella* virulence can be affected by a temperature-sensing protein TlpA (Hurme and Rhen, 1998). In addition, cell growth phases can affect *Salmonella* virulence. A recent study in the *flhDC* promoter region of the *Salmonella* flagellar regulon reported that the *flhDC* transcription regulators are growth phase dependent, with the maximum expression in the mid-log cultures (Mouslim and Hughes, 2014). Thus, mid-log cultures of *S*. Typhimurium Δ*yqiC* used in our study might induce the phenotypic effect of *yqiC* on bacterial colonization and invasion, which was inhibited by overnight cultures and low temperature.

*Salmonella* employs *yqiC* to suppress type-1 fimbriae overexpression for manipulating other pathogenic factors, such as flagella and motility, in bacterial colonization and invasion in the human intestinal epithelium. In general, bacterial adherence and internalization are two major characteristics of *Salmonella* involved in bacterial colonization during the interactions between *Salmonella* and host cells. Type-1 fimbriae are major mediators during *Salmonella* biofilm formation (Boddicker et al., 2002). However, the deletion of *yqiC* activated type-1 fimbrial expression on the bacterial surface and biofilm formation, but led to loss of its colonization and invasion capability in all four human cell lines in our study. Thus, *yqiC* is required for *Salmonella* colonization and invasion in host cells through other pathogenic factors regulated by the type-1 fimbrial expression instead of the structural fimbrial expression on the bacterial surface and biofilm formation. Our data meant that the up-regulated type-1 fimbriae was not the crucial factor in *yqiC* mediated bacterial colonization. The colonization effect of type-1 fimbriae-like appendages on the *S*. Typhimurium surface and biofilm formation was probably overwhelmed by the augmented effect of flagella and SPI-1.

**FIGURE 8:**
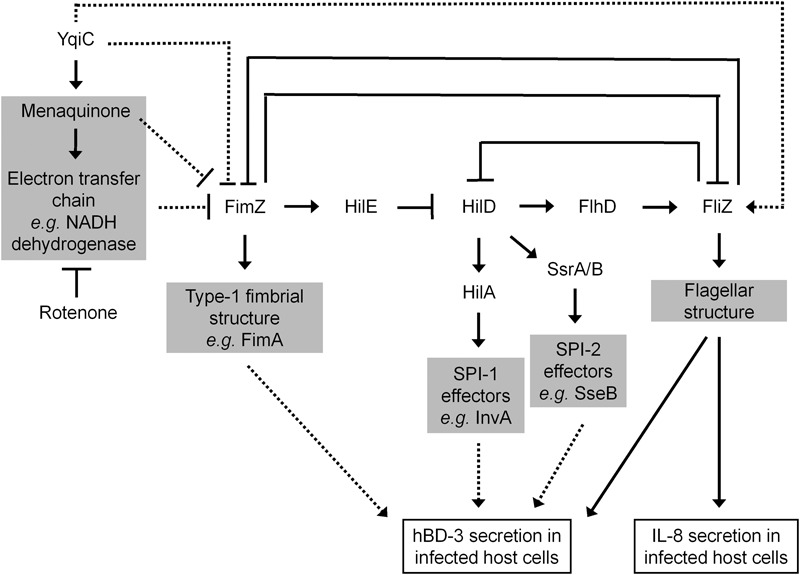
**Scheme of the *yqiC* regulatory network in *Salmonella*.** This scheme summarizes the role of *yqiC* in the regulation of type-1 fimbrial, SPI-1, SPI-2, and flagellar genes. YqiC enhances menaquinone biosynthesis and its related electron transport chain, negatively controls FimZ and its downstream regulatory network, and positively manipulates FliZ and its subsequent flagellation. Our data supported that YqiC can suppress the expression of type-1 fimbriae in *Salmonella*. The activation of the type-1 fimbrial regulator FimZ can inhibit the expression of flagellar, SPI-1, and SPI-2 genes by inhibiting the regulators HilD, HilA, SsrA/B, and FliZ, resulting in the suppression of the host immune responses, including the expression of IL-8 and hBD-3. The solid and dotted lines indicate well-documented regulations and putative connections in the regulator network, respectively.

*yqiC* is involved in *S*. Typhimurium flagellation and motility, both of which are downregulated by the overexpression of the FimZ-centered type-1 fimbriae regulatory circuit. By contrast, *yqiC* can switch off this *S*. Typhimurium circuit for maintaining bacterial virulence factors, such as flagella, SPI-1, and SPI-2 effectors. Our results exhibited that *yqiC* deletion in *Salmonella* reduces the production of structural flagella on the bacterial outer membrane, thus reducing bacterial motility. Type-1 fimbrial expression in *Salmonella* is regulated by positive regulators FimZ and FimY (Yeh et al., 1995). *fimZ* controls type-1 fimbriae production and mediates bacterial motility. However, it is unclear whether *fimY* is correlated with bacterial motility. In addition, *fimZ* positively controls *hilE*, which negatively regulates *hilD*, an activator of *hilA*, to upregulate the expression of the SPI-1 gene *invA* (Baxter and Jones, 2005). FimZ appears to be the dominant activator of the P*fimA* promoter to regulate the expression of the *fim* structural genes. FimY activates FimZ expression and also activates its own expression slightly. FimZ alone can enhance the expression of *hilE*, a repressor of SPI-1 gene expression. Moreover, activated FimZ generates hyperfimbriated but non-motile *S.* Typhimurium in soft agar, and such non-motility correlates with the downregulation of the *flhDC* master flagellar operon (Clegg and Hughes, 2002). FliZ, a flagellar regulator, can inhibit the expression of the type-1 fimbrial gene through post-transcriptional regulation of FimZ (Saini et al., 2010). Therefore, FimZ can be considered a key molecule between flagellar and fimbrial formation in *S*. Typhimurium. In addition, *hilD* of *Salmonella* is involved in the regulation of SPI-2 gene expression (Martinez et al., 2011). The expression of FimZ and type-1 fimbriae in *Salmonella* gradually reduces after infecting murine macrophages, and the constitutive expression of FimZ and type-1 fimbriae can suppress the SPI-2 gene activation (Wang et al., 2013). Furthermore, the type-1 fimbrial regulator FimZ can control *Salmonella* motility by inactivating the *flhDC* flagellar operon (Clegg and Hughes, 2002). Our qRT-PCR analysis revealed that the expression of the SPI-1 gene *invA*, the SPI-2 gene *sseB*, and the flagellar gene *flhD* was downregulated in *S.* Typhimurium Δ*yqiC*. Bridging the existing knowledge, *Salmonella yqiC* can have a vital role in the orchestration of the type-1 fimbrial expression on flagella, SPI-1, and SPI-2, possibly through FimZ and FliZ (**Figure 8**).

Both *yqiC* and *fliC* are required for host IL-8 production in human IECs after *S*. Typhimurium infection. Bacterial flagellin is considered the main ligand of *Salmonella* that elicits inflammatory responses, such as IL-8 production, in host cells (McCormick et al., 1993; Hayashi et al., 2001). In this study, IL-8 and hBD-3 secretion from LS174T cells reduced after *S.* Typhimurium Δ*yqiC* infection compared with that of *S.* Typhimurium SL1344, and the reduced IL-8 secretion was non-significantly higher on *S.* Typhimurium Δ*yqiC* infection than on *S.* Typhimurium Δ*fliC* infection (**Figure 2A**). These findings suggest that the non-SPI gene *yqiC* is a *Salmonella* virulence genetic factor for induction of host cell inflammation and is at least as influential as *fliC*. The significantly attenuated colonization and invasion of *S.* Typhimurium Δ*yqiC*, resulting in fewer intracellular bacteria compared with the wild-type strain, can partly clarify the decreased IL-8 secretion from the infected host cells. Meanwhile, such attenuated host inflammation can be attributed to the effects of other virulence factors affected by *yqiC* deletion, including downregulation of the SPI-1 and SPI-2 genes *invA* and *sseB* through upregulation of *fimZ* in *S.* Typhimurium Δ*yqiC* (**Figure 6**). Furthermore, the reduced expression of structural flagella in *S.* Typhimurium Δ*yqiC* (**Figure 3**), observed in this study, further addresses the contribution of *yqiC* to *Salmonella* virulence.

Both *yqiC* and *fliC* are responsible for hBD-3 production in LS174T cells after *S.* Typhimurium infection. This is the first study to report that *S.* Typhimurium can trigger hBD-3 secretion in human IECs. hBD-3 expression is significantly higher in normal tonsils and skin, but absent in the small intestine (Harder et al., 2001). Thus far, avian studies have reported contrasting results associated with β-defensin-3 expression. AvBD-3 expression is enhanced in uninfected broiler chicken guts (Ramasamy et al., 2012), and its gene expression is upregulated in the bone marrow and spleen after *Salmonella* infection (Ma et al., 2013). By contrast, AvBD-3 expression is significantly upregulated in the uninfected chicken ovaries, but not in the *Salmonella*-infected chicken ovaries (Ebers et al., 2009; Michailidis et al., 2012). Our study by using hBD-3-expressing LS174T cells presented further evidence that hBD-3 is constitutively secreted in the uninfected human intestinal epithelium and that the expression can be significantly increased by *S*. Typhimurium infection. Similar to the induction of human hBD-2 expression in Caco-2 cells (Ogushi et al., 2001; Takahashi et al., 2001), our study demonstrated that hBD-3 production can be induced by *Salmonella* flagellin, and *yqiC* has a role in the regulatory network involving type-1 fimbriae, SPI-1, SPI-2, and flagella (**Figure 8**). We hypothesize that in addition to flagellar genes, the expression of type-1 fimbrial, SPI-1, or SPI-2 genes is involved in the regulation of hBD-3 expression in host cells, and additional studies are warranted for clarification.

For the first time, we demonstrated that *Salmonella yqiC* accounts for menaquinone biosynthesis within bacterial cells and has a key role in flagellation and suppression of type-1 fimbrial expression, similar to the effects of NADH dehydrogenase in the electron transport chain. Menaquinone is one of the isoprenoid quinines that are located in the inner membrane of bacteria and function as electron and proton carriers in the respiratory electron transport chain and as antioxidants (Nowicka and Kruk, 2010). Most gram-negative facultative anaerobic rods, such as *E. coli* and *Klebsiella pneumoniae*, contain mixtures of menaquinones, demethylmenaquinones, and ubiquinones (electron carriers in the electron transport chain), with the menaquinones and demethylmenaquinones being the major quinone types (Collins and Jones, 1981). However, the composition of quinone pools in *Salmonella* remains unclear. In bacteria, menaquinone-carried electrons can be bound with the oxidized reductants such as NADH, succinate, formate, and hydrogen, whereas menaquinones can be oxidized by coupling them with reduction of oxidants such as oxygen, nitrogen dioxide, nitrite, and sulfate. Thus, the redox loop mechanism or the respiratory chain drives the proton pumps for ATP synthesis (Lancaster and Simon, 2002). In addition, the flagellar export apparatus of *Salmonella* comprising FlhA, FlhB, FliO, FliP, FliQ, and FliR exerts its ATPase activity at the cytoplasmic side, where ATP is hydrolyzed as the energy source to drive flagellin exportation for the assembly of each flagellum (Minamino et al., 2014). In *E. coli*, the respiratory electron transport system correlates with flagella formation (Hertz and Bar-Tana, 1977). In *Salmonella*, the ubiquinone synthetic pathway is required for flagellar biosynthesis (Bar-Tana et al., 1980). However, the mechanism underlying menaquinone-controlled flagellation in *Salmonella* remains unclear. *ubiB*, is probably involved in menaquinone or ubiquinone biosynthesis in *Salmonella*, and can suppress type-1 fimbrial expression because *ubiB* deletion in *Salmonella* enhances type-1 fimbrial expression (Chuang et al., 2008). These findings are consistent with our result that absence of menaquinone activated the expression of type-1 fimbriae, and defective flagellation coexisted in S. Typhimurium Δ*yqiC*. In this study, complementation with menaquinone in *S*. Typhimurium Δ*yqiC* confirmed that menaquinone is required for type-1 fimbrial, flagellar, SPI-1, and SPI-2 gene expression when *yqiC* is disrupted in *S*. Typhimurium. Therefore, menaquinone is a critical mediator within *Salmonella* affected by *yqiC* to negatively control *fimZ* expression. Meanwhile, activated *fimZ* expression can reduce flagellar biosynthesis and SPI-1 and SPI-2 gene expression (**Figure 8**). Because of the lack of menaquinone inhibitors, we could not confirm whether menaquinone downregulates *fimZ* expression directly or through the electron transport chain. Menaquinone analogs can be used as inhibitors to inhibit the growth of *Staphylococcus aureus, B. anthracis, Streptococcus pyogenes*, and *Streptococcus agalactiae* (Schlievert et al., 2013). In this study, we used an NADH dehydrogenase inhibitor, rotenone, in *S*. Typhimurium, and its effects on the regulation of type-1 fimbrial and flagellar gene expression, but not on the regulation of SPI-1 and SPI-2 gene expression, were similar to those of *yqiC* deletion. Rotenone only affects NADH dehydrogenase activity in complex I of the electron transport chain (Ueno et al., 1994), but succinate dehydrogenase and nitrate reductase complexes are also responsible for electron transport in bacteria (Hurdle et al., 2011). Therefore, the regulation of SPI-1 and SPI-2 gene expression by *Salmonella yqiC* correlates with other enzymes, rather than NADH dehydrogenase, in the electron transport chain. Furthermore, YqiC of *S*. Typhimurium shares approximately 25% amino acid homology with IbpA and IbpB of *E. coli*, as per our preliminary alignments. IbpA and IbpB, the heat shock proteins of *E. coli*, are correlated with *E. coli* resistance to oxidative stress induced by hydrogen peroxide and copper (Kitagawa et al., 2000; Matuszewska et al., 2008). Thus, *yqiC* might facilitate cell damage repair under oxidative stress through menaquinone and its related processes in the electron transport chain. This claim warrants further investigation.

## Conclusion

*yqiC* significantly contributes to *Salmonella* colonization and invasion as well as host inflammation and innate immunity after infection. Our study provides additional novel knowledge regarding the presence of an upstream virulence gene manipulating *fimZ*-dominated type-1 fimbriae regulation and its downstream virulence factors, flagella, SPI-1, and SPI-2. We observed that the electron transport chain, including its functioning compound menaquinone, is possibly involved in regulation of these crucial *Salmonella* virulence factors. Therefore, the evidence suggests that *yqiC* is a promising target gene for developing novel antimicrobials and immunomodulators against salmonellosis.

## Author Contributions

K-CW performed the research and wrote the article; C-HH, S-MD, and C-KC analyzed data; H-WF and M-TH performed the technique of molecular biology, and S-BF designed the research and assisted correction of the article.

## Conflict of Interest Statement

The authors declare that the research was conducted in the absence of any commercial or financial relationships that could be construed as a potential conflict of interest.

## Footnotes

1 http://www.ncbi.nlm.nih.gov/tools/primer-blast/

## Abbreviations

AMP: antimicrobial peptide
AvBD: avian β-defensin
CFU: colony-forming unit
*E. coli*: *Escherichia coli*
ELISA: enzyme-linked immunosorbent assay
FBS: fetal bovine serum
*fim*: Type-1 fimbriae
hBD: human β-defensin
HPLC: high-performance liquid chromatography
IEC: intestinal epithelial cell
IL: interleukin
MAPK: mitogen-activated protein kinase
MQ: menaquinone
NADH: nicotinamide adenine dinucleotide (NAD) + hydrogen (H)
NF-κB: nuclear factor kappa-light-chain-enhancer of activated B cells
PBS: phosphate buffered saline
qRT-PCR: quantitative real-time polymerase chain reaction
*S*. Typhimurium: *Salmonella enterica* serovar Typhimurium
SPI: *Salmonella* pathogenicity island
T1SSs: type I secretion systems
T3SSs: type III secretion systems
TEM: transmission electron microscopy
TLC: thin-layer chromatography
